# Household Hints and Recipes

**Published:** 1885-01

**Authors:** 


### Household Hints & Recipes.

To Remove Paint Spots From Clothing.—Saturate the fabric with equal parts of turpentine and water of ammonia.

Galvanized iron pails are not desirable receptacles for drinking water. The zinc coating is apt to be affected by the water and an oxide of zinc formed.

Tar, to Remove.—Tar may be removed from the hands by rubbing with the outside of fresh orange or lemon peel and drying immediately. The volatile oils dissolve the tar so that it can be rubbed off.

Invisible Ink.—Linseed oil, one part; water of ammonia, twenty parts; pure water, one hundred parts. Mix thoroughly, and shake well before using. To make the writing appear, dip the paper in water. The characters fade as the paper dries.

Compound Tincture of Benzoin for Chilblains.—Dr. N. C. B. Haveland, in the *Australian Chemist and Druggist*, states that compound tincture of benzoin, if freely and frequently applied to the inflamed parts, seldom fails to alleviate and finally cure chilblains.

To Prevent Sore Throat.—According to the *Medical Record*, a gargle made of strong black tea and used cold, night and morning, is now the fashionable preventive in London against falling a victim to sore throat during the cold winds of the spring.

Oil Cloth, to Clean.—Oil cloth may be improved in appearance by rubbing it with a mixture of a half-ounce of beeswax in a saucerful of turpentine. Set this in a warm place until they can be thoroughly mixed. Apply with a flannel cloth and then rub with a dry flannel.

Polish for Bronze, Brass and Silver.—Fifty parts of cocoanut soap cut up in small pieces are gently heated with sufficient water to form a pasty mass, to which is added a mixture consisting of 5 parts tripoli and 1½ parts ammonium carbonate. The polish is put up in stone jars covered with parchment paper.—*Leitmeritzer Rundschau.*

Steel Lacquer.—A lacquer for steel may be made of 10 parts of clear mastic, 5 of camphor, 15 of sandarac, and 5 of elemi gums dissolved in pure alcohol filtered and applied cold. This varnish is transparent.

Porcelain Finish.—White paint, suitable for reflectors, may be made by mixing dry white zinc carbonate with silicate of potash liquid. After each coat, artificial heat should be employed to hasten the drying.

Cracks in Floors.—A very complete filling for open cracks in floors may be made by thoroughly soaking newspapers in paste made of one pound of flour, three quarts of water, and a tablespoonful of alum, thoroughly boiled and mixed. Make the final mixture about as thick as putty, and it will harden like *papier mache.*

To Clean Gold.—Dull gold can be cleaned with the following solution: 80 grammes each of calcium hypochlorite and sodium bicarbonate, and 20 grammes of common salt in 3 quarts of distilled water. Preserve in corked bottles. Articles to be cleaned should be put into a basin and covered with the mixture. After a time they should be taken out, washed, rinsed in alcohol, and dried in sawdust. The articles will then be as good as new.

Furniture Polish.—The subjoined simple preparation will be found desirable for cleaning and polishing old furniture: Over a moderate fire put a perfectly clean vessel; into this drop two ounces of white or yellow wax; when melted, add four ounces of pure turpentine, then stir until cool, when it is ready for use. The mixture brings out the original color of the wood, adding a lustre equal to that of varnish. By rubbing with a piece of fine cork, it may, when it fades, be removed.

To Clean Chamois.—Chamois may be cleaned in a weak solution of soda in warm water. Rub plenty of soft soap into the leather, and allow it to soak for two hours. Then rub it well until it is quite clean, and rinse it well in a weak solution composed of soda, yellow soap, and warm water. If rinsed in water only, it becomes hard when dry, and unfit for use. After rinsing, wring it well in a coarse towel, and dry quickly; then pull it about and brush it well, and it will become softer and better than most new leathers.

HARD PLASTER CASTS.—To a pint of milk of lime add ten to fifteen drops of liquid silicate of soda (water-glass), then mix in plaster of Paris until the consistency of a thick cream. This will set in about five minutes.

---

RUBBER RINGS.—When the rubber rings used for closing preserve jars become so hard as to be useless, their elasticity may be restored by leaving them for half an hour in two parts of water of ammonia and one of water.

---

TO CLEAN MARBLE.—Mix powdered chalk and powdered pumice stone, each one part, with two parts carbonate of soda into a paste with water, and rub it thoroughly on the marble, or mix quick-lime and strong soap lye to the consistency of milk, and apply it to the marble for twenty-four hours. In either case wash off thoroughly with soap and water.

---

POCKET GLUE.—

Best glue, 12 ozs.
Sugar, 5 ozs.
Water, sufficient.

Soak the glue in water over night and dissolve it by heat in the smallest possible quantity of water. Add the sugar to the hot solution, and dry the composition like jujube paste, in oiled moulds.

---

SUBSTITUTE FOR GROUND GLASS.—The best substitute I have found for an emergency, or in fact for constant use, while clean, is to take a piece of "white lead putty," equal parts white lead and common putty—that can be had in almost any town—work them together until quite soft, make into a ball and roll over the surface of clean glass, and you have a ground glass just as fine as you choose to make it, and that will answer all purposes.—*Photography.*

---

CINDERS IN THE EYE.—One of the minor trials in railway travel arises from cinders in the eye. A simple and effective cure may be found in one or two grains of flaxseed, which can be placed in the eye without pain or injury. As they dissolve, a glutinous-substance is formed, which envelopes any foreign body that may be under the lid and the whole is washed out. A dozen of these seeds should constitute a part of every traveler's outfit.—*Popular Science News.*

FURNITURE POLISH.—One of our correspondents sends us the following formula, which, he says, is easily prepared and works well:

Alcohol, 4 oz.
Turpentine, 2 oz.
Damar Varnish, 1 oz.
Linseed Oil (raw), 8 oz.
Acetic Acid, 1 oz.

---

TO DISTINGUISH BUTTERINE.—The following simple method has been suggested for approximately judging of the purity of a specimen of butter.

Melt the butter, and then cool it as rapidly as possible by means of some ice-cylinder put into it. Lard, which is a copious constituent of butterine, will sink to the bottom and any genuine butter present will rise, while there will be a distinctly visible zone or line of contact between the two.

---

CEMENT FOR CONNECTING GLASS AND BRASS.—Puscher, in the *Chemiker Zeitung*, states that the following cement resists kerosene, and is useful for cementing the brass collars to glass lamps:

Caustic soda, 1 part.
Resin, 3 parts.
Water, 5 parts.

Boil together. The resin soap thus produced is mixed and well kneaded with half its weight of plaster of Paris. It hardens in about three-quarters of an hour. If zinc white or dry white lead is used, it hardens more slowly.

---

COLORING SOLDER.—The following is a method for coloring soft solder so that when it is used for uniting brass the colors may be about the same: First, prepare a saturated solution of sulphate of copper—blue-stone—in water, and apply some of this on the end of a stick to the solder. On touching it then with an iron or a steel wire it becomes coppered, and by repeating the experiment the deposit of copper may be made thicker and darker. To give the solder a yellow color, mix one part of a saturated solution of sulphate of zinc with two of sulphate of copper; apply this to the coppered spot, and rub it with a zinc rod. The color can be still further improved by applying gilt powder and polishing. On gold jewelry, or colored gold, the solder is first coppered as above, then a thin coat of gum or isinglass solution is laid on and bronze powder dusted over it, making a surface which can be polished smooth and brilliant after the gum is dry.

